# Carbonic Anhydrase III Is Expressed in Mouse Skeletal Muscles Independent of Fiber Type-Specific Myofilament Protein Isoforms and Plays a Role in Fatigue Resistance

**DOI:** 10.3389/fphys.2016.00597

**Published:** 2016-12-15

**Authors:** Han-Zhong Feng, J.-P. Jin

**Affiliations:** Department of Physiology, Wayne State University School of MedicineDetroit, MI, USA

**Keywords:** carbonic anhydrase III, skeletal muscle fatigue, fast and slow twitch muscle fibers, myofilament protein isoforms, *TNNT1* myopathy

## Abstract

Carbonic anhydrase III (CAIII) is a metabolic enzyme and a regulator for intracellular pH. CAIII has been reported with high level expression in slow twitch skeletal muscles. Here we demonstrate that CAIII is expressed in multiple slow and fast twitch muscles of adult mouse independent of the expression of myosin isoforms. Expressing similar fast type of myofilament proteins, CAIII-positive tibial anterior (TA) muscle exhibits higher tolerance to fatigue than that of CAIII-negative fast twitch extensor digitorum longus (EDL) muscle in *in situ* contractility studies. We further studied the muscles of CAIII knockout (*Car3*-KO) mice. The loss of CAIII in soleus and TA muscles in *Car3*-KO mice did not change muscle mass, sarcomere protein isoform contents, and the baseline twitch and tetanic contractility as compared with age-matched wild type (WT) controls. On the other hand, *Car3*-KO TA muscle showed faster force reduction at the beginning but higher resistance at the end during a fatigue test, followed by slower post fatigue recovery than that of WT TA muscle. Superfused *Car3*-KO soleus muscle also had faster total force reduction during fatigue test than that of WT soleus. However, it showed a less elevation of resting tension followed by a better post fatigue recovery under acidotic stress. CAIII was detected in neonatal TA and EDL muscle, downregulated during development, and then re-expressed in adult TA but not EDL muscles. The expression of CAIII in *Tnnt1*-KO myopathy mouse soleus muscle that has diminished slow fiber contents due to the loss of slow troponin T remained high. *Car3*-KO EDL, TA, and soleus muscles showed no change in the expression of mitochondria biomarker proteins. The data suggest a fiber type independent expression of CAIII with a role in the regulation of intracellular pH in skeletal muscle and may be explored as a target for improving fatigue resistance and for the treatment of *TNNT1* myopathies.

## Introduction

Carbonic anhydrases (CA) catalyze the reversible hydration of CO_2_ to H_2_CO_3_. At least 16 CA isozymes have been identified in mammals with different tissue distribution and catalytic activity (Imtaiyaz Hassan et al., 2013). CAIII is an ~30-kDa cytosolic protein (Carter et al., 1978) present at high levels in liver, adipocytes, and skeletal muscles (Sly and Hu, 1995). It is a low activity enzyme among CA isozymes (Koester et al., 1977, 1981) but is resistant to most sulfonamide inhibitors (Sanyal et al., 1982). The physiological function of CAIII is controversial. CAIII expression is negligible in preadipocytes and becomes abundant after differentiation (Lynch et al., 1993), implicating a role in fatty acid metabolism (Lyons et al., 1991). CAIII may facilitate rapid conversion of glycolytic intermediates to oxaloacetate and citrate and stimulate their incorporation into fatty acids. However, adipocyte CAIII expression in obese mice is lower than that in lean mice (Lynch et al., 1992).

CAIII expression in skeletal muscle was observed as fiber type-specific, mainly reported in type I slow-twitch muscle fibers (Shima, 1984; Vaananen et al., 1985; Frémont et al., 1988; Zheng et al., 1992; Sly and Hu, 1995). In mouse, CAIII transcripts are first detected in the myotomes of somites in embryos between 9.5 and 10.5 days post coitum, and gradually increase in all skeletal muscles during the next 4 days of development (Lyons et al., 1991). After birth, CAIII mRNAs are expressed at high level in mature slow muscle fibers. The expression of CAIII during early muscle development suggests a correlation with skeletal muscle differentiation. However, *Car3* gene knock-out (*Car3*-KO) in mice did not affect normal development, growth and life span with minimum phenotype in soleus muscle that has a high slow fiber content (Kim et al., 2004). Studies of the gastrocnemius muscle of *Car3*-KO mice using *in situ* ^31^P magnetic resonance detected reductions of phosphocreatine and ATP, elevations of ADP and inorganic phosphate, and a decrease of pH during 2 min of fatigue contractions, which were at significantly higher degrees than that of wild type (WT) controls (Liu et al., 2007).

A large number of sarcomeric protein mutations have been found to cause inherited cardiomyopathies (Watkins et al., 2011), including many in the gene encoding cardiac troponin T (TnT) (Willott et al., 2010; Sheng and Jin, 2014). In contrast, very few myopathic mutations are identified in skeletal muscle isoforms of TnT. The most investigated skeletal muscle TnT mutations are five nemaline myopathies alleles in *TNNT1* gene encoding the slow skeletal muscle isoform of TnT. *TNNT1* myopathies are featured by loss of slow twitch muscle fibers and presented with severe muscle atrophy, weakness and failure of respiratory muscle (Johnston et al., 2000; Jin et al., 2003; Amarasinghe et al., 2016). Mouse models of *TNNT1* myopathy reproduced the slow muscle atrophy and degeneration phenotypes and showed a significant loss of fatigue resistance of soleus and diaphragm muscles (Feng et al., 2009; Wei et al., 2014).

To investigate the potential function of CAIII in skeletal muscle and in adaptation to the loss of slow fibers in *TNNT1* myopathy, here we demonstrated that CAIII is expressed in multiple slow and fast twitch muscles of adult mouse independent of the expression of myosin isoforms. Expressing similar myofilament protein contents, tibial anterior (TA) expressing a high level of CAIII exhibits higher resistance to fatigue than that of CAIII-negative extensor digitorum longus (EDL) muscle. *Car3*-KO TA muscle showed faster force reduction at the beginning but higher resistance at the end, followed by a slower post fatigue recovery than that of WT TA muscle. *Car3*-KO soleus muscle also had faster total force reductions during fatigue but less elevation of resting tension followed by a better post fatigue recovery under acidotic stress. The expression of CAIII in *Tnnt1*-KO myopathy mouse soleus muscle that has diminished slow fibers remained high. These data suggest a fiber type independent expression of CAIII with a role in the regulation of intracellular pH and fatigue resistance in skeletal muscle cells.

## Materials and methods

### Animal models

*Car3*-KO mice (Kim et al., 2004) and matching strain (129SEVE) wild type mice were purchased from Jackson Lab. The development of *Tnnt1*-KO mice in C57BL/6 strain was reported previously (Wei et al., 2014). The genotypes of the mice were verified by PCR. Mice were housed in the animal facility on a 12:12-h light-dark cycle (6:00 AM/6:00 PM) and fed a standard pellet diet and water. Two to three month old mice were used for functional studies. Previous studies of *Car3*-KO mice did not find gender-generated differences (Kim et al., 2004). The comparison of male and female data in our present study did not indicate statistical significances in muscle weight, force production and fatigability. Therefore, pooled data from male and female mice were used for soleus muscle studies. The TA and EDL muscle studies were from male mice. All animal protocols are approved by the Institutional Animal Care and Use Committees of Wayne State University. The expression or lack of CAIII and slow skeletal muscle TnT in the muscles studied were confirmed by Western blot as described below.

### SDS-PAGE and western blotting

Fresh or frozen muscle tissues were rapidly homogenized in SDS-PAGE sample buffer containing 2% SDS and 1% β-mercaptoethanol, pH8.8, using a high speed mechanical homogenizer (Pro 250, Pro Scientific Inc.) to extract total proteins. After heating at 80°C for 5 min, the samples were clarified by centrifugation at 14,000 × g in a microcentrifuge for 5 min. Protein samples were resolved on 14% SDS-gel with acrylamide:bisacrylamide ratio of 180:1 or 12% SDS-gel with acrylamide:bisacrylamide ratio of 29:1 in a modified Laemmli buffer system in which both stacking and resolving gels were at pH 8.8. The protein bands in the gel were visualized by staining with Coomassie Blue R 250. Total protein in each lane was quantified by ImageJ software for normalizing the amount of sample loading.

Copies of the SDS-gels were transferred to nitrocellulose membrane using a Bio-Rad semidry electrotransfer device at 5 mA/cm^2^ for 15 min. The blotted membranes were blocked in 1% bovine serum albumin (BSA) in Tris-buffered saline (TBS, 150 mM NaCl, 50 mM Tris, pH 7.5) with shaking at room temperature for 30 min. The blocked membrane was probed with an anti-CAIII monoclonal antibody (mAb) CP3 (Jin et al., 1996), an anti-TnI mAb TnI-1 (Jin et al., 2001), an anti-TnT mAb 2C8 (Jin and Chong, 2010), or antibodies against peroxisome proliferator-activated receptor coactivator 1 (PGC-1) (ab54481, Abcam) and voltage-dependent anion channel (VDAC) (4866S; Cell Signaling Technology, Beverly, MA), diluted in TBS containing 0.1% BSA, with gentle rocking at 4°C overnight. The membranes were then washed three times with TBS containing 0.5% Triton X-100 and 0.05% SDS, incubated with alkaline phosphatase-labeled goat anti-mouse IgG second antibody (Santa Cruz Biotechnology), washed again as above, and developed in 5-bromo-4-chloro-3-indolyl phosphate/nitro blue tetrazolium substrate solution to visualize the protein bands detected.

### Glycerol-SDS-PAGE

Myosin heavy chain (MHC) isoforms expressed in muscle tissues were examined using glycerol-SDS-PAGE (Feng et al., 2011). Briefly, SDS-PAGE samples equivalent to 5 μg of muscle tissue (wet weight) were resolved on 8% polyacrylamide gel with acrylamide:bis-acrylamide ratio of 50:1, prepared in 200 mM Tris base, 100 mM glycine, pH 8.8, containing 0.4% SDS and 30% glycerol. The stacking gel contained 4% polyacrylamide with acrylamide:bis-acrylamide ratio of 50:1, 70 mM Tris-HCl (pH 6.7), 4 mM EDTA, 0.4% SDS, and 30% glycerol. The upper cathode running buffer consists of 100 mM Tris base, 150 mM glycine, 0.1% SDS, and 10 mM β-mercaptoethanol. The lower anode running buffer was 50% dilution of the upper running buffer without β-mercaptoethanol. The 0.75-mm-thick Bio-Rad minigels were run at 100 V in an icebox for 24 h. The resolved protein bands were visualized after staining with Coomassie blue R250.

### *In situ* measurement of muscle contractile functions

Due to the large size of TA muscle, *ex vivo* superfusion may generate hypoxia in the center of the muscle due to limited diffusion of oxygen. Therefore, muscle contractility was measured *in situ* with physiological blood supply to compare TA and EDL muscle functions.

Mice were anesthetized by inhalation of 3.5% isoflurane for induction and 2% isoflurane for maintenance using a small animal anesthesia system (SomnoSuite, Kent Scientific Corp). On a temperature controlled platform (Aurora Scientific, Aurora, Ontario, Canada) and under a heating lamp to maintain the body temperature at 37 ± 0.5°C using PhysioSuite system (Kent Scientific Corp.), hair was removed from the leg area, the distal tendon of TA or EDL muscle was exposed surgically and made partially free for mounting to a force transducer (300C-LR, Aurora Scientific Corp) through a stainless steel wire hook and a serrated clip that was able to hold the tendon tightly. As adult mouse TA muscle generates more than 100 g force which is out of range for the force transducer, the hook was connected to a short point of the lever arm to expand the range of measurements. The actual force was then calibrated to correct for the shorter length of the lever arm. The proximal end of tibial bone was mounted on the platform with a pair of pointed screws. The foot was taped a position that the muscle was aligned with the force transducer.

The sciatic nerve was exposed and freed carefully avoiding injury. A pair of custom-made platinum wire electrodes was placed around the nerve for applying stimulations using an electrical stimulator (Aurora Scientific Corp). Continuing dripping of warm Kreb's buffer bubbled with 95% O_2_, 5% CO_2_ at 37°C around the exposed muscle tendon was used to prevent tissue drying. The exposed sciatic nerve was also covered by a filter paper wetted with dripping Kreb's buffer. Biphasic square pules of 0.1 ms duration at a voltage 50% above the threshold were applied to stimulate twitch contractions of the muscle. After 20 min equilibration at 0.05 Hz twitch contractions, the resting length of muscle was slowly increased to reach an optimal resting length that generated maximal isometric force. Then the muscle was kept at the optimal resting length for tetanic contraction, fatigue, and recovery studies.

After 20 min equilibration with tetanic contractions at 300 Hz for 300 ms in every 1 min, a series of frequencies (200–380 Hz) were tested to identify the optimum frequency that produced maximum tetanic force, in which EDL and TA muscles showed the same optimal frequency of 300 Hz to produce maximum force. Stimulation of 300 ms duration in every 1500 ms at the optimal frequency was applied for 300 repeats to induce muscle fatigue. One minute after the fatigue contractions, 20 min of 300 ms tetanic contractions every 1 min was recorded for the recovery of muscle contractility.

### Histology

After *in situ* contractility measurement, the TA, EDL, and soleus muscles of the other leg was exposed. Two 30-G needles were inserted into the proximal end of the muscle and the distal tendon. The distance between two needles was measured when the ankle was at 90° angle. The muscle was then removed with the needles attached and placed in optimal cutting temperature compound (O.C.T.) in a cryostat tissue holder with the tendons pinned down onto a cork at the *in situ* muscle length between the two needles. The muscle tissue in O.C.T. was dipped in pre-cooled isopentane at −160°C and freeze for 1 min before submerged in liquid nitrogen. This protocol eliminates ice crystal formation inside muscle fibers. Five or ten-micron cryo-sections were cut, processed for hematoxylin and eosin staining, and imaged using a Zeiss Axio Observer A1 microscope with an attached digital camera.

### Immunohistochemical staining of muscle sections

As previously reported (Wei et al., 2014), cross sections of mouse soleus muscle were blocked in phosphate-buffered saline (PBS) containing 0.05% Tween-20 (PBS-T) and 1% BSA at room temperature for 30 min. Endogenous peroxidase was inactivated by incubation with 1% H_2_O_2_ in PBS-T at room temperature for 10 min. After a wash with PBS-T, the muscle sections were incubated with an anti-MHC I mAb FA2 (Jin et al., 1990) or SP2/0 myeloma ascites control in PBS-T containing 0.1% BSA at 4°C overnight. After washes with PBS-T to remove excess primary antibodies, the sections were incubated with horseradish peroxidase conjugated anti-mouse second antibody in PBS-T containing 0.1% BSA at room temperature for 1 h. After washes with PBS-T to remove excess second antibody, MHC I expression in type I fibers was visualized via 3,3-diaminobenzidine-H_2_O_2_ substrate reaction after developing in a dark box for 30 s. The reaction was terminated by washes with 20 mM Tris-HCl, pH 7.6. Nuclei were then counterstained with Haematoxylin for 5 min followed by washes with distilled water. The muscle sections were mounted in PBS containing 50% glycerol, sealed using Cytoseal, and photographed using a Zeiss Observer 125 microscope.

### Measurement of contractile function of superfused soleus muscle at normal and acidotic PH

Acidosis generated with lowering the pH of perfusion buffer was employed to investigate the mechanism for CAIII to alter fatigue resistance under stress conditions. *Ex vivo* contractility measurement of intact soleus muscle was performed with superfusing the muscle under the carbogen equilibration with different levels of CO_2_. Using a protocol modified from our previous studies (Feng et al., 2011), intact soleus muscle was carefully isolated with both tendons avoiding stretching damage and mounted vertically to a dual-mode lever arm force transducer (300B, Aurora Scientific) in an organ bath containing 100 mL modified Kreb's solution (118 mM NaCl, 25 mM NaHCO_3_, 4.7 mM KCl, 1.2 mM KH_2_PO_4_, 2.25 mM MgSO_4_, 2.25 mM CaCl_2_, and 11 mM D-glucose, continuously gassed with 95% O_2_, 5% CO_2_, pH 7.4). Maintained at 25 ± 0.5°C in the bath with thermos-controlled circulating water jacket, contractions were elicited with bipolar pulse field electrical stimulation using a stimulator (701B, Aurora Scientific). Twitch contractions were elicited with supramaximal pulses (0.1 ms, 28 V/cm), unless specified otherwise. Tetanic contractions were elicited with a train of the same pulses at 100 Hz for 0.7 s. Isometric force data were collected via a digital controller A/D interface (604C, Aurora Scientific) and recorded using Chart software (ASI, Aurora Scientific). Developed twitch and tetanic forces were determined at the optimal muscle length that gave the highest twitch force and calculated by subtracting the resting tension from the total force.

After 20-min equilibration with 0.7 s tetanic contractions per minute, various stimulation frequencies were tested to determine the optimal frequency that produced maximum tetanic force. A 300 s fatigue protocol was performed with intermittent tetani of 700 ms every second. One minute after the end of fatigue, recovery of muscles contractility was recorded for 20 min with 0.7 s tetanic contractions every 1 min. After the baseline study, the muscle was re-equilibrated, and the perfusion buffer was switched to 70% O_2_, 30% CO_2_ to apply acidosis and repeat the fatigue and recovery protocol.

### Data analysis and statistics

Densitometry analysis of SDS-gel and Western blots was done using ImageJ software on images scanned at 600 dpi resolution. The force of EDL and TA muscles was normalized to muscle weight that represents the total volume of contractile units, i.e., the sarcomeres, instead of muscle cross sectional area since the organization for muscle fibers in EDL and TA is rather different. Quantitative data are presented as mean ± SE, and statistical analysis was performed using student's *t*-test or two-way ANOVA as noted in the figure legends. A Bonferroni *post hoc* follow-up test was conducted to compare the mean values between WT and *Car3*-KO groups.

## Results

### Broad expression of CAIII in mouse skeletal muscles

**Figure 1:**
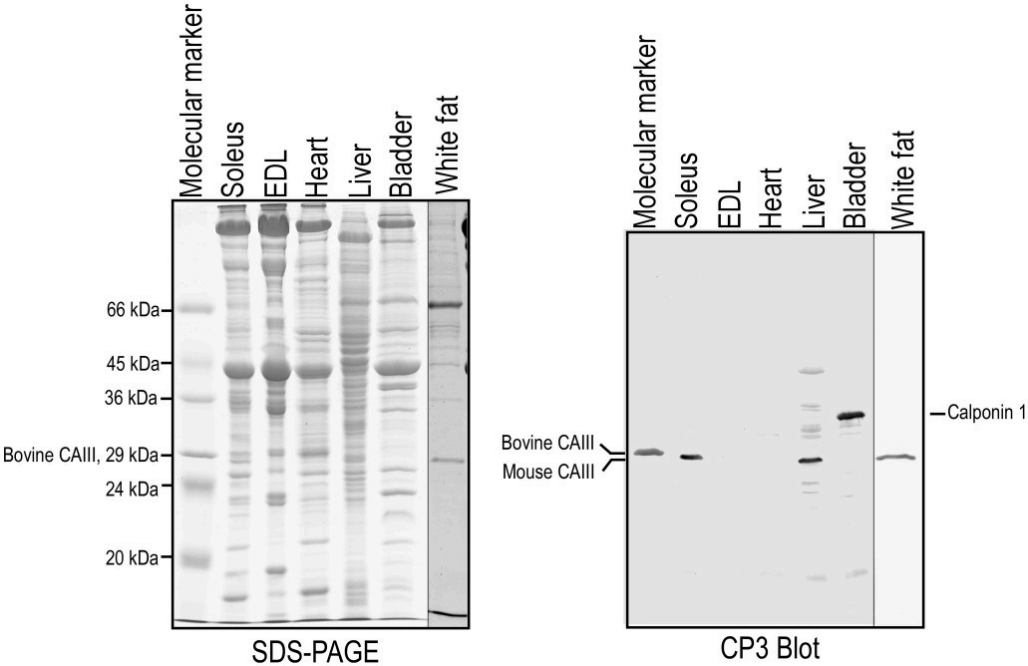
**An mAb recognizing CAIII**. SDS-PAGE and Western blotting showed that CP3, an mAb originally raised against smooth muscle specific calpon in 1 (Jin et al., 1996), strongly and specifically recognizes bovine CAIII (one of the commercial SDS-gel molecular weight markers in the L70 set from Sigma) and CAIII in mouse liver, white fat and soleus muscle. Mouse bladder, heart, and fast twitch skeletal muscle EDL were negative as expected.

The CP3 mAb that we previously developed by immunization using chicken smooth muscle calponin 1 (Jin et al., 1996) strongly reacts with purified bovine CAIII included in the Sigma molecular weight marker set L70 (Figure 1). Further testing showed that CP3 specifically recognizes mouse CAIII in liver, white fat and slow type muscle such as the soleus but not EDL. As expected, no CAIII was detected by CP3 in mouse heart and bladder smooth muscle (Figure 1). While the specific epitope structure shared by calponin 1 and CAIII merits a future study, mAb CP3 provides a useful tool to study the expression of CAIII in skeletal muscles.

**Figure 2:**
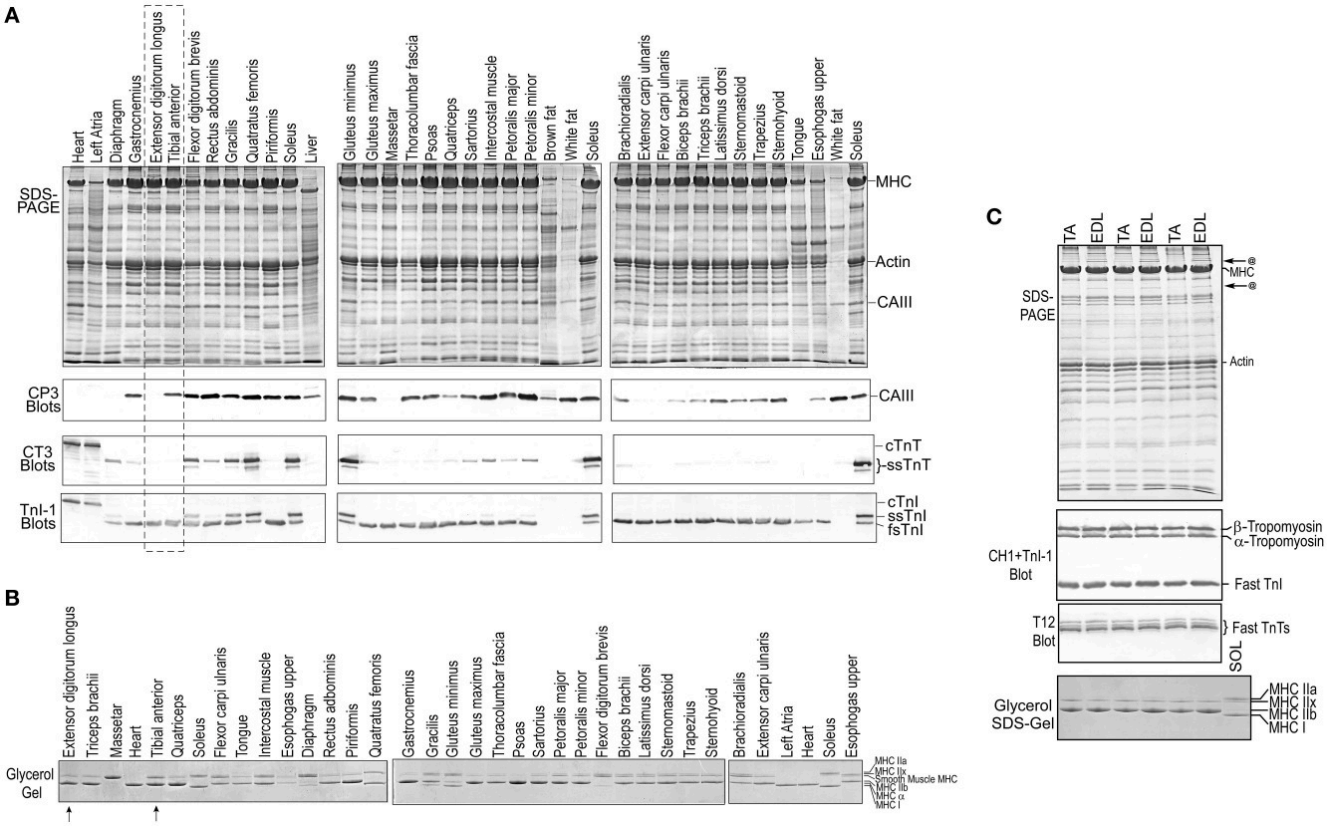
**Broad expression of CAIII in mouse skeletal muscles. (A)** SDS-PAGE and mAb CP3 Western blots of multiple adult mouse skeletal muscles showed that CAIII is expressed at high levels in slow fiber-containing muscles and as well as in many pure fast fiber muscles. The relative slow and fast fiber contents of the muscle were estimated from the level of slow TnT detected in the mAb CT3 blots and the ratio of slow and fast isoforms of TnI in the mAb TnI-1 blots. **(B)** Glycerol-SDS-PAGE showed various MHC isoform contents in the fast fiber type mouse skeletal muscles studied without clear correlation with the expression of CAIII. **(C)** CAIII positive fast muscle TA and CAIII negative fast muscle EDL were chosen for functional comparisons.

Multiple adult mouse skeletal muscles were examined with Western blotting using mAb CP3 (Figure 2). The accompanying Western blots of mAb TnI-1 that recognizes all three isoforms of TnI demonstrated the relative fast and slow fiber contents in these muscles (Figure 2). The results in Figure 2A demonstrated that all slow fiber-rich muscles where slow TnI is detected at significant levels and most of the pure fast fiber mouse muscles in which only fast TnI is present expressed high or significant amounts of CAIII. Only a few fast fiber skeletal muscles are negative, including EDL, masseter, tongue and the upper portion of esophagus (as well as the heart). The glycerol-SDS-gel data shown in Figure 2B further demonstrate that the expression of myosin isoforms in the fast fiber skeletal muscles has no definitive correlation to the expression of CAIII. The observation that CAIII expression in skeletal muscle is independent of the fiber type-specific myofilament proteins and its expression in many but a few fast twitch muscles intrigued our investigation on the physiological significance.

### Distinguishable contractility and fatigue resistance of mouse TA and EDL muscles *in situ*

**Figure 3:**
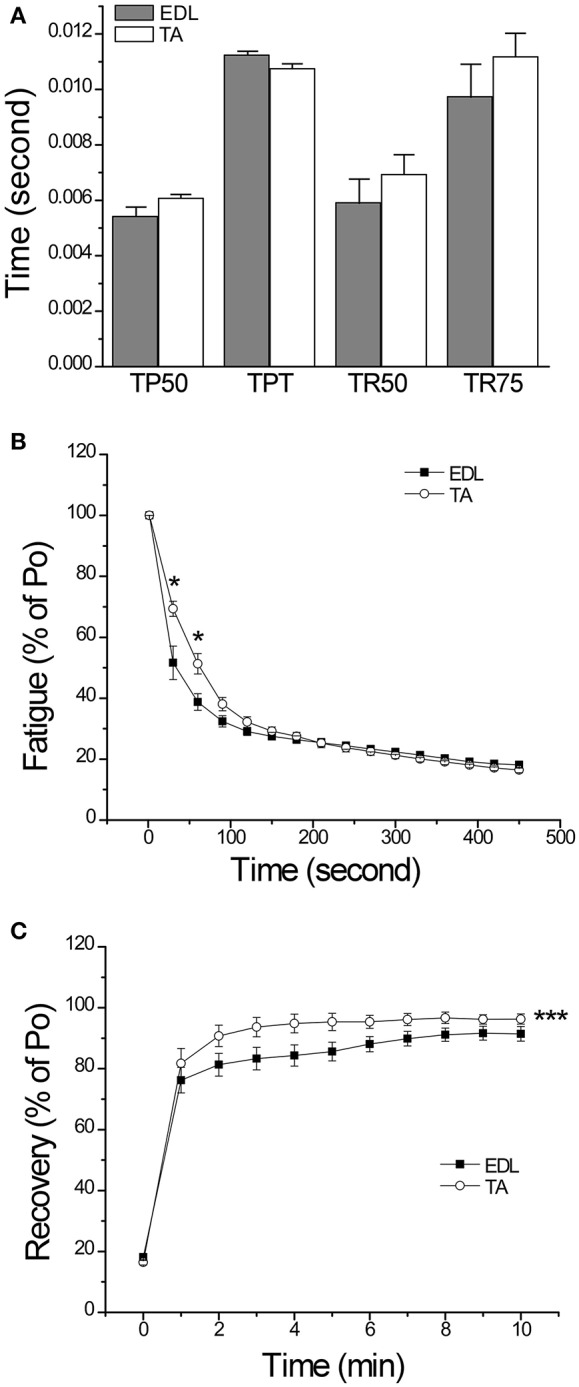
*****In situ*** contractility and fatigue resistance of mouse EDL and TA muscles. (A)** The time parameters of twitch contraction and relaxation (TP50, time to develop 50% peak force; TPT, time to develop peak force; TR50, time for 50% relaxation; and TR75, time to 75% relaxation) were not significantly different in EDL and TA muscles. **(B)** Force normalized to the pre-fatigue maximum force in *in situ* fatigue study showed that CAIII-positive TA muscle had a slower decrease in contractility than that of CAIII-negative EDL muscle reflecting a higher fatigue resistance. **(C)** TA muscle also showed a better post fatigue recovery than that of EDL. The data are presented as mean ± SE. *N* = 4 mice each in TA and EDL groups. Statistical analysis was performed using Student's *t*-test. ^*^P < 0.05 vs. EDL using Student's *t*-test. ^***^P < 0.001 vs. EDL using two-way ANOVA test with Bonferroni adjustment (for TA vs. EDL: *DF* = 1, *F* = 27.61, *P* = 1.56 × 10^−6^).

The finding that CAIII can be either totally absent or expressed at a significant level in some pure fast fiber mouse muscles (Figure 2A) provided us with informative experimental systems for functional investigation. TA and EDL muscles are anatomically adjacent fast twitch limb muscles, of which a significant level of CAIII is present in TA but completely absent in EDL (Figure 2C), indicating a representative pair to study the function of CAIII in TA muscle. While the difference in fiber orientations makes their muscle weight-normalized forces non-comparable, *in situ* muscle contractility studies demonstrated that TA and EDL muscles had no difference in the time parameters of twitch contraction and relaxation (Figure 3A). The 300 s *in situ* muscle fatigue protocol then revealed a slower force drop in TA muscle than that of EDL during the first 100 s (Figure 3B). TA muscle also exhibited a better post fatigue recovery than that of EDL (Figure 3C). These results suggest that the presence of CAIII in TA muscle may have contributed to the higher resistance to fatigue than that of the CAIII-negative EDL muscle.

### *Car3*-KO decreases the fatigue resistance of mouse TA muscle

**Figure 4:**
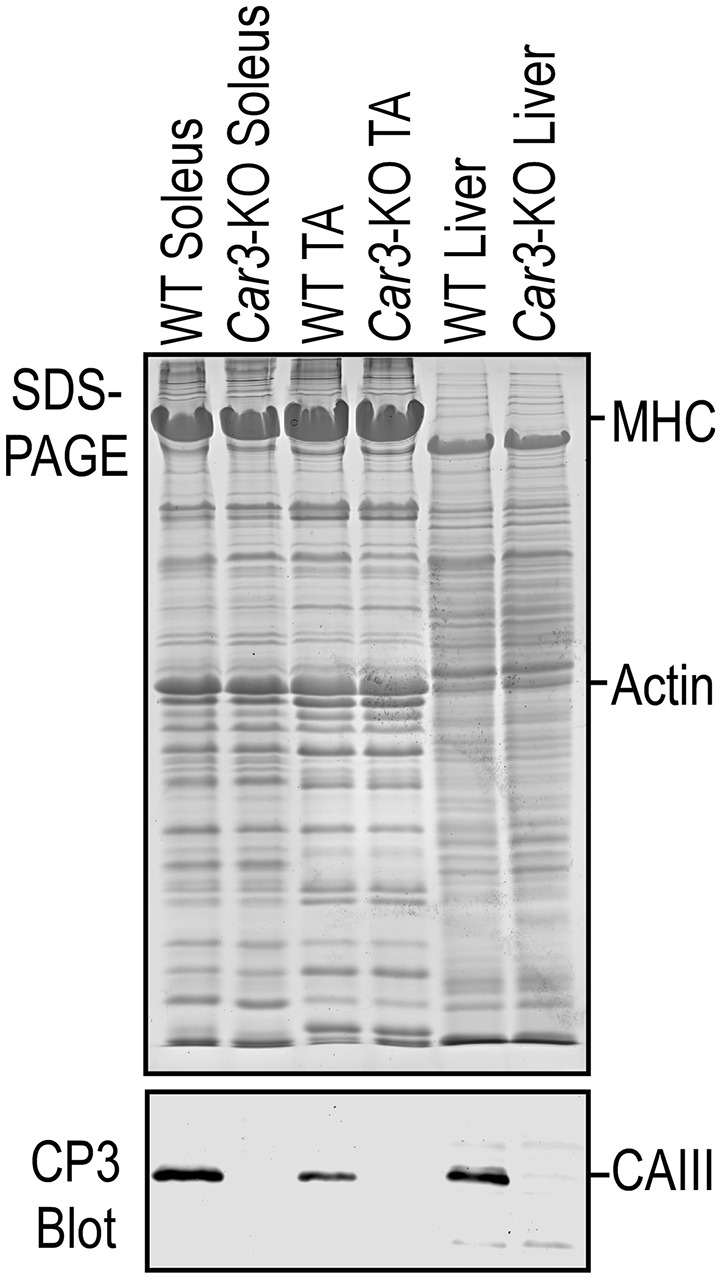
**Deletion of CAIII in skeletal muscle of ***Car3***-KO mice**. SDS-PAGE and mAb CP3 Western blot with WT controls confirmed the loss of CAIII in *Car3*-KO mouse soleus and TA muscles and liver.

To further investigate the function of CAIII in TA muscle, *Car3*-KO mouse muscles were studied. mAb CP3 Western blot confirmed the absence of CAIII in *Car3*-KO mouse TA muscle and liver (Figure 4). Body weight of 2-month old *Car3*-KO mice showed no difference from WT control (Supplement Figure 1). Normalized to body weight, the weights of TA and EDL muscles were similar in *Car3*-KO and WT mice (Supplement Figure 1). Histology examination found no signs of inflammation or fiber degeneration in the TA muscle of young *Car3*-KO mice as compared with WT control (Supplement Figure 1). The cross sectional area of TA muscles of Car3-KO and WT mice are also similar (Supplement Figure 1). These data demonstrate that the loss of CAIII did not cause atrophy or degenerative defect in the TA muscle of *Car3*-KO mice. The EDL and soleus muscles of *Car3*-KO mice also did not show atrophy or degenerative changes (data not shown).

**Figure 5:**
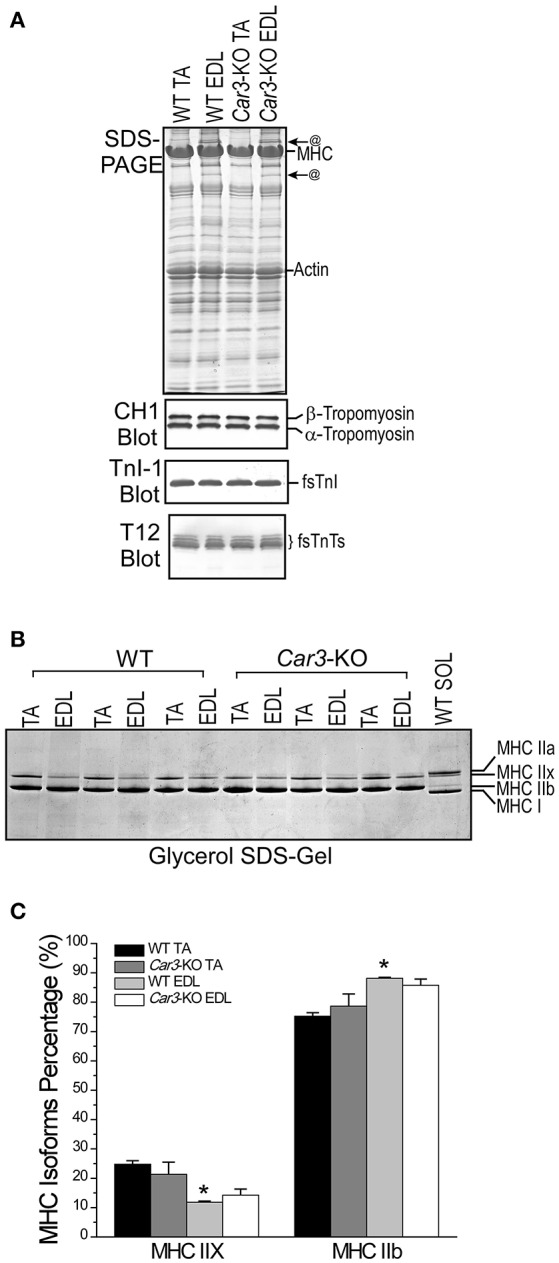
**Adult ***Car3***-KO and WT mouse TA muscles express similar myofilament protein isoforms. (A)** SDS-PAGE and Western blots showed that the overall protein profile and the expression of tropomyosin and troponin isoforms in *Car3*-KO TA or EDL muscles were not different from the WT control. The SDS-gel showed two protein bands in EDL but not TA muscle (@), which remain to be identified. **(B)** Glycerol-SDS-gel showed that MHC isoforms expressed in *Car3*-KO TA and EDL muscles are also similar to their WT counterparts. **(C)** Quantification of MHC IIx and IIb in EDL and TA of WT and *Car3*-KO mice showed that MHC IIx was higher and MHC IIb lower in WT TA than that in WT EDL muscles (^*^P < 0.05). *Car3*-KO did not cause significant change in the levels of MHC IIx and IIb in TA or in EDL muscles. The data are presented as mean ± SE. *N* = 3 mice in each groups. Statistical analysis was performed using Student's *t*-test.

The SDS-PAGE gel and Western blots in Figure 5A showed that adult mouse TA and EDL muscles had very similar overall protein profiles except for two visible protein bands in EDL but not TA. The functional significance of these unknown proteins needs further investigation. The Western blots in Figure 5A showed similar isoform expression patterns of tropomyosin, fast TnI and splice forms of fast TnT in TA and EDL muscles. No significant change in tropomyosin, TnT, TnI and MHC isoforms in *Car3*-KO TA muscle as compared with that of WT TA. Glycerol-SDS-gel and densitometry quantification in Figures 5B,C showed that MHC isoforms IIx and IIb are expressed in these two muscles. The only detectable difference her is that the level of MHC IIx is higher and MHC IIb lower in TA than that in EDL muscles, a trend remained in the *Car3*-KO muscles.

**Figure 6:**
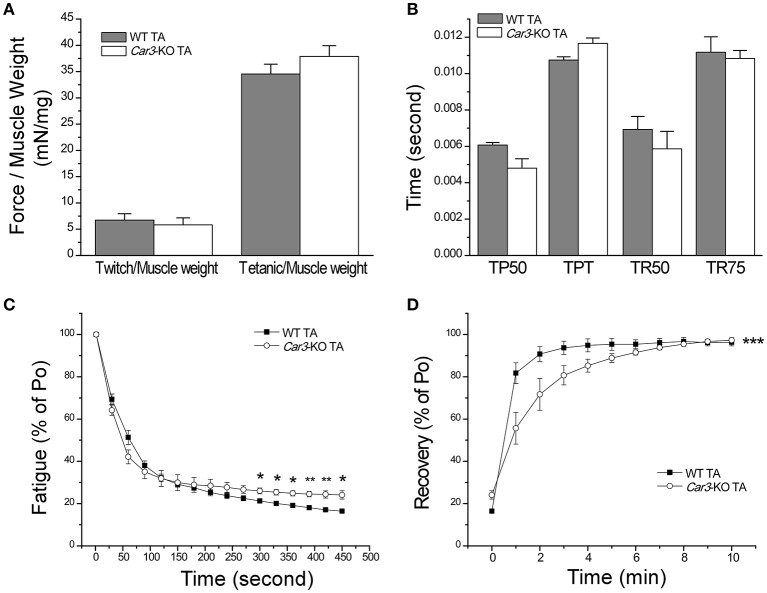
**Higher fatigue resistance but slower post fatigue recovery of ***Car3***-KO TA muscle than WT controls. (A)** Normalized to muscle weight, *in situ* twitch and tetanic forces were similar in *Car3*-KO and WT TA muscles. **(B)** *Car3*-KO and WT TA muscles had similar time parameters for twitch contraction and relaxation (TP50, time to develop 50% peak force; TPT, time to develop peak force; TR50, time for 50% relaxation; and TR75, time to 75% relaxation). **(C)** *In situ* fatigue protocol showed higher fatigue resistance of *Car3*-KO vs. that of WT TA muscles. **(D)** However, *Car3*-KO muscle had slower post-fatigue recovery than WT control whereas the maximum level of recovery was not affected. The data are presented as mean ± SE. *N* = 4 mice in WT and *n* = 3 in *Car3*-KO groups. ^*^P < 0.05 and ^**^P < 0.01 vs. WT using Student's *t*-test. ^***^P < 0.001 vs. WT using two-way ANOVA test and adjusted mean comparison using Bonferroni test (for the level between WT and *Car3*-KO: *DF* = 1, *F* = 1 6.04, *P* = 1.76 × 10^−4^).

*In situ* contractility studies showed that TA muscles of *Car3*-KO and WT mice had similar twitch and tetanic forces normalized to muscle weight (Figure 6A) and similar contractile time parameters (Figure 6B), demonstrating similar baseline functions. *In situ* fatigue protocol revealed that *Car3*-KO TA muscle exhibited a faster initial decrease of contractile force although the remaining contractile force at the end of the fatigue protocol was higher as compared to that of WT TA muscle (Figure 6C). Consistent with the possible contribution of CAIII to higher resistance to fatigue of TA muscle (Figure 3C), *Car3*-KO TA muscle showed a significantly slower recovery than that of WT control (Figure 6D). The results support the role of CAIII in the fatigue resistance of TA muscle.

### *Car3*-KO increased the resistance of mouse soleus muscle to acidosis

**Figure 7:**
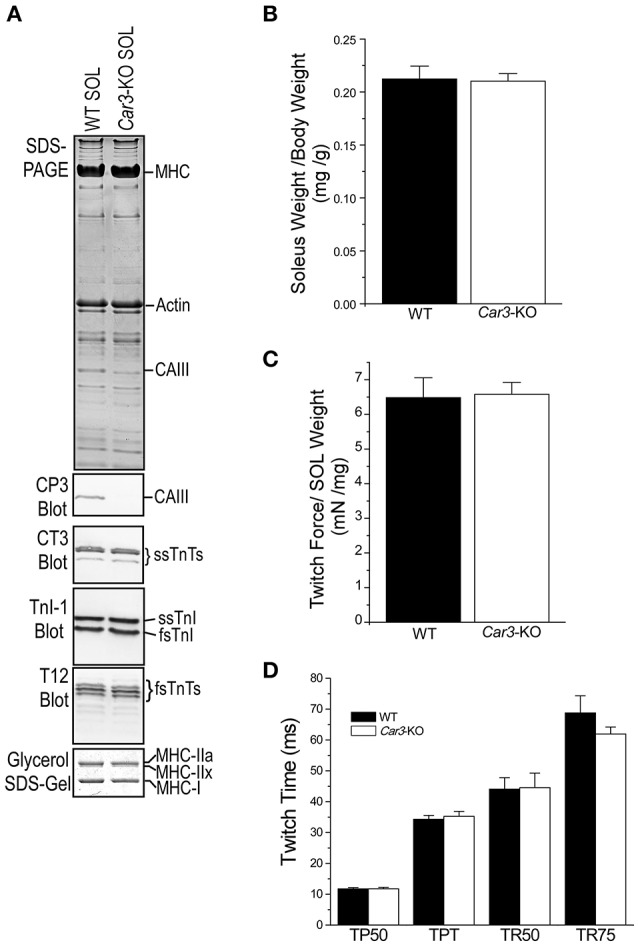
**Similar expression of myofilament protein isoforms, total mass and contractility of ***Car3***-KO and WT mouse soleus muscle**. **(A)** SDS-PAGE, Western blots and glycerol-SDS-gel showed no difference between *Car3*-KO and WT mouse soleus muscles in the overall protein profiles and the expression of troponin and MHC isoforms. **(B)** No difference was found in the soleus muscle weight to body weight ratio between *Car3*-KO and WT mice. **(C)** Twitch force of superfused soleus muscle normalized to muscle weight was similar in *Car3*-KO and WT groups. **(D)** *Car3*-KO and WT soleus muscles showed similar time parameters of twitch contraction and relaxation (TP50, time to develop 50% peak force; TPT, time to develop peak force; TR50, time for 50% relaxation; and TR75, time to 75% relaxation). The data are presented as mean ± SE. *N* = 6 mice each in WT and *Car3*-KO groups. Statistics analysis was performed using Student's *t*-test.

To investigate the role of CAIII in the fatigue resistance of skeletal muscle via regulating the intracellular pH environment, we compared contractility of superfused soleus muscles of *Car3*-KO and WT mice under normal and acidotic conditions. SDS-gel and Western blot analysis showed that *Car3*-KO soleus muscle had no change in TnI, TnT and MHC isoform contents as compared with that of WT soleus (Figure 7A). Normalized to body weight, the soleus muscle masses of *Car3*-KO, and WT mice were also similar (Figure 7B). The maintained muscle fiber type and mass in *Car3*-KO soleus muscle justifies its use in the study of CAIII's role in pH regulation and fatigue resistance.

**Figure 8:**
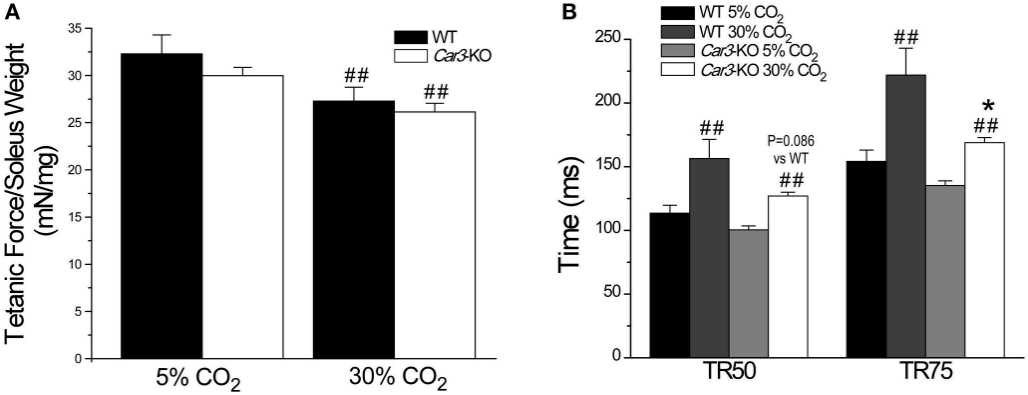
*****Car3***-KO prevents the acidosis-caused prolongation of relaxation in mouse soleus muscle. (A)** *Car3*-KO and WT soleus muscles showed no significant difference in tetanic force production at both normal extracellular pH (5% CO_2_), which decreased by similar degrees at acidotic extracellular pH (30% CO_2_). **(B)** Acidotic extracellular pH produced a significant prolongation of the time for 50% and 75% relaxation (TR50 and TR75) post tetanic contraction in WT soleus muscle. *Car3*-KO minimized this acidosis-caused slowdown of relaxation. The data are presented as mean ± SE. *N* = 6 mice each in WT and *Car3*-KO groups. ^*^P < 0.05 vs. WT in Student's *t*-test. ^##^*P* < 0.01 vs. 5% CO_2_ in paired Student's *t*-test.

Superfused at normal pH (7.4) using Kreb's buffer gassed with 5% CO_2_, *Car3*-KO and WT soleus muscles showed no difference in twitch force (Figure 7C), and time parameters of contractile and relaxation (Figure 7D). At this physiological condition, the tetanic contractile force of *Car3*-KO and WT soleus muscles were similar (Figure 8A). When using Kreb's buffer equilibrated with 30% CO_2_ to lower the extracellular pH to 6.5, tetanic contraction force of *Car3*-KO and WT soleus muscles decreased in similar degrees (Figure 8A). Whereas, the relaxation time of tetanic contraction was prolonged in both *Car3*-KO and WT soleus muscles at the acidotic condition, it was significantly less severe in *Car3*-KO soleus (Figure 8B).

**Figure 9:**
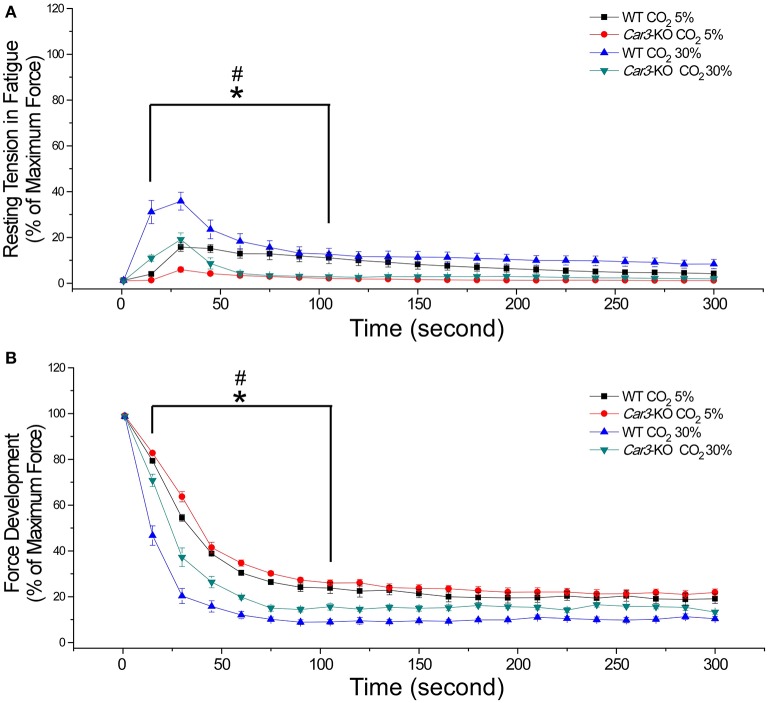
**Deletion of CAIII in mouse soleus muscle minimizes the rise of resting tension during fatigue contractions under acidosis stress. (A)** During 300 s tetanic contractions, there was a rise of resting tension in the first 100 s. Acidotic extracellular pH (30%CO_2_) significantly augmented this rise in WT soleus muscle, which was minimized in *Car3*-KO muscle. **(B)** Corresponding with the rise of resting tension, active tetanic tension development dropped rapidly during the first 100 s of fatigue contractions, most severe in WT soleus muscle at acidotic pH, which was minimized in *Car3*-KO muscle. The data are presented as mean ± SE. *N* = 6 mice each in WT and *Car3*-KO groups. ^*^P < 0.05 vs. WT. ^#^*P* < 0.05 vs. 30%CO_2_. Statistical test was performed using two-way ANOVA and adjusted mean comparison of Bonferroni test between the four groups (for WT and *Car*3-Ko with 5% and 30% CO_2_, *DF* = 3, *F* = 165.4 and P was close to 0).

It was interesting to note that during the fatigue test, the resting tension of soleus muscle rose in the early phase. This occurred in both WT and *Car3*-KO soleus muscles and at higher levels at the acidotic condition (Figure 9A). Consistent with the effect of deleting CAIII on minimizing the impairment of relaxation time in acidosis, *Car3*-KO soleus muscle exhibited much less increase in resting tension as compared with that of WT soleus, especially at the acidotic condition (Figure 9A). Correspondingly, the development force calculated by subtracting resting tension from the total force in each contraction was better maintained in *Car3*-KO soleus muscle than WT control during the fatigue test (Figure 9B). Supporting the observation that this may be a specific effect in acidosis, *in situ* contractility and fatigue experiments showed that under physiological *in vivo* pH, *Car3*-KO, and WT mouse TA and EDL muscles both had no increase in resting tension (Supplement Figure 2).

This finding suggests that the role of CAIII in increasing fatigue resistance is compromised under acidotic conditions. Therefore, CAIII may function in fatigue resistance of skeletal muscle via a bi-phasic regulation of intracellular pH. Under normal pH, it increases resistance to fatigue whereas in acidosis it has an opposite effect. The bi-phasic fatigue responses of *Car3*-KO TA muscle during *in vivo* fatigue test shown in Figure 6C support this hypothesis by the beneficial effect in the later phase of fatigue contractions when local acidosis may have occurred.

### Postnatal down-regulation and re-expression of CAIII in adult mouse TA muscle

**Figure 10:**
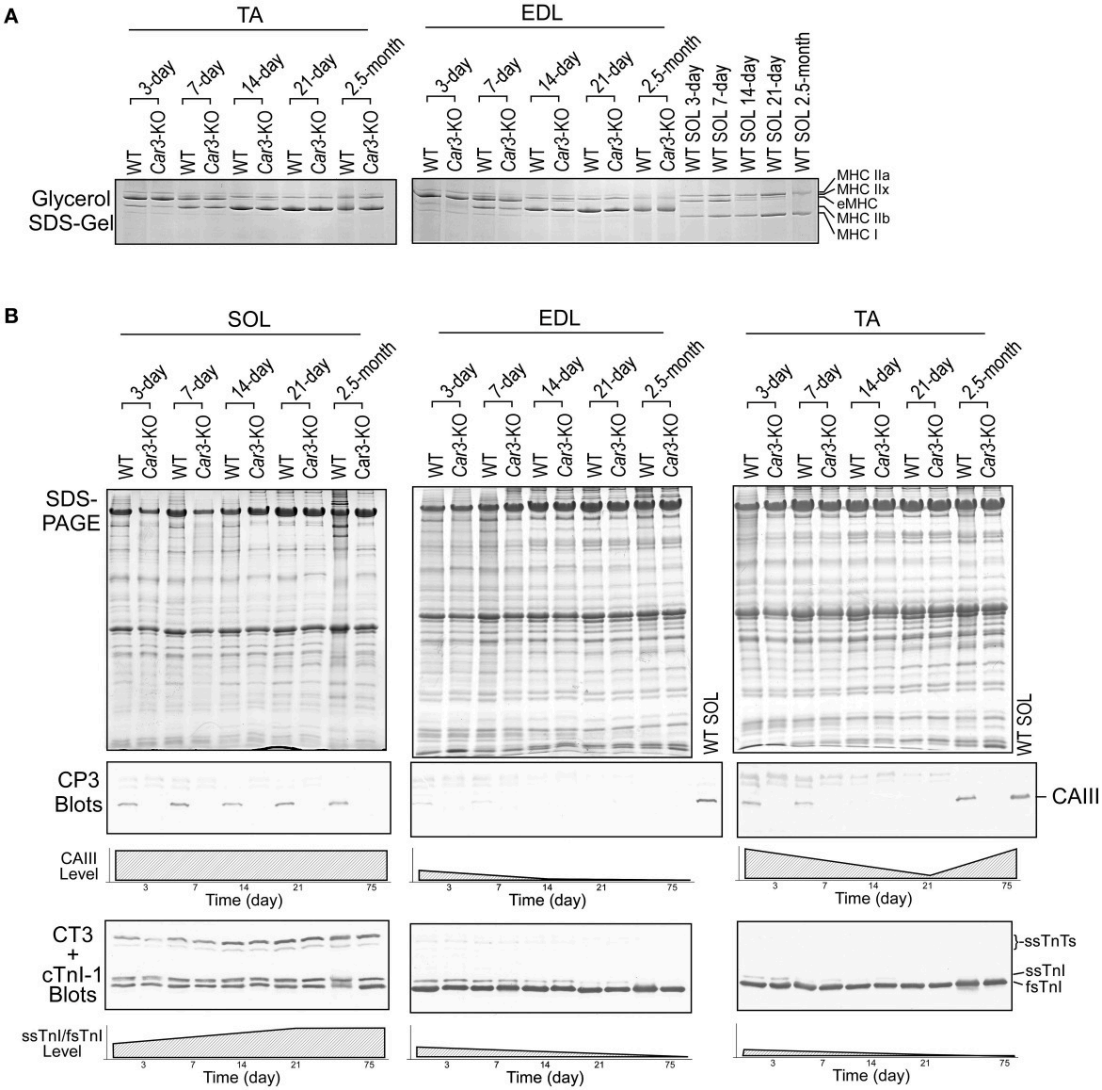
**CAIII expression in neonatal and adult mouse TA, EDL and soleus muscles. (A)** Glycerol-SDS-gel showed that MHC isoforms had similar contents and patterns of developmental switch in *Car3*-KO and WT TA and EDL muscles. **(B)** SDS-gels and Western blots showed that CAIII was expressed in neonatal soleus, TA and EDL muscles. The expression slightly lowered in soleus and ceased in EDL muscles during postnatal development. The expression of CAIIl in TA muscle diminished during postnatal development but re-activated in young adult. The expression of slow vs. fast troponin isoforms during postnatal development was examined using mAbs CT3 and TnI-1 Western blots and no difference was found between *Car3*-KO and WT muscles.

Glycerol-SDS-gel electrophoresis showed no difference in the developmental switching of MHC isoforms between *Car3*-KO and WT TA and EDL muscles (Figure 10A). Western blots showed that CAIII was detectable in neonatal soleus, EDL and TA muscles and continuously expressed in soleus muscle (Figure 10B). The expression of CAIII in EDL muscle ceased after 7 days postnatal. The expression of CAIII in TA muscle showed similarly postnatal down-regulation but was re-activated to a significant level in adult as shown in the 2.5-month-old sample (Figure 10B). The developmental isoform switches of thin filament regulatory proteins TnI and TnT were not different in *Car3*-KO and WT TA muscles. This observation implicates that the postnatal expression of CAIII in fast fiber skeletal muscles may be an adaptation to specific functions.

### Maintained expression of CAIII in *Tnnt1* myopathy mouse soleus muscle

**Figure 11:**
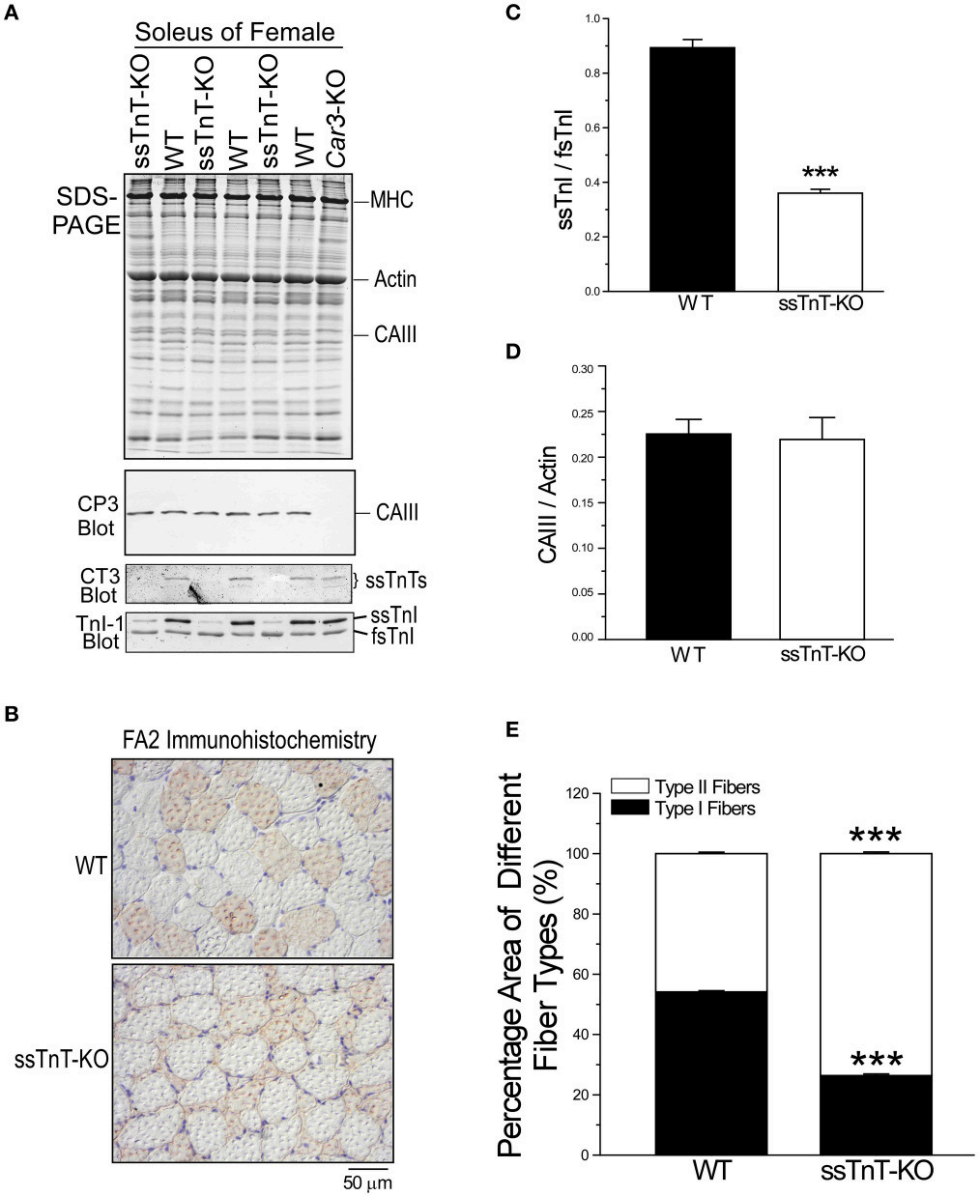
**Expression of CAIII in soleus muscle of a mouse model of ***Tnnt1*** myopathy. (A)** The expression level of CAIII was examined in adult soleus muscle of WT and *Tnnt1*-KO (ssTnT-KO) mice. The Western blot and glycerol-SDS-gel demonstrate the loss of slow TnT in *Tnnt1*-KO soleus muscle and the switches to significantly higher contents of fast types of myofilament proteins (the decreases in ssTnI/fsTnI ratio). Despite the diminished slow fiber content, the level of CAIII expression was remained. **(B)** Immunohistochemistry staining using anti-MHC I mAb FA2 of cross sections of *Tnnt1*-KO soleus muscle showed atrophy of type I slow fibers and hypertrophy of type II fast fibers as compared with WT control. **(C)** Densitometry quantification of the TnI-1 mAb Western blots confirmed the significantly decreased ratio of ssTnI/fsTnI in *Tnnt1*-KO soleus muscle in comparison with that of WT soleus muscle. **(D)** Densitometry quantification of Western blot normalized to the actin band in SDS-gel confirmed no decrease of CAIII in *Tnnt1*-KO soleus muscle vs. the WT control. **(E)** Quantification of the percentage cross sectional areas of type I and type II fibers in soleus muscles confirmed the atrophy of type I fibers and hypertrophy of type II fibers in *Tnnt1*-KO mice vs. that in WT mice. The data are shown as mean ± SE. *N* = 3 mice each in WT and *Tnnt1*-KO groups. ^***^P < 0.001 as compared with WT using Student's *t*-test.

The expression of CAIII was examined in soleus muscle of WT and *Tnnt1*-KO myopathy mice (Johnston et al., 2000; Jin et al., 2003; Wei et al., 2014). Western blot and glycerol-SDS-gel demonstrate that the loss of slow TnT in *Tnnt1*-KO mice caused a drastic loss of slow type I fibers in soleus muscle indicated by the diminished levels of slow TnI as compared to WT controls (Figures 11A,C), reflecting a switch to more fast fiber content. Despite the diminished slow fiber contents, the level of CAIII was maintained in *Tnnt1*-KO soleus muscle (Figures 11A,D). Together with the increased content of fast fibers (Figures 11B,E), the results support a notion that the expression of CAIII is not restricted to slow fibers but corresponding to the contractile features of the soleus muscle.

### No significant change in mitochondria function in CAIII KO mouse muscles

**Figure 12:**
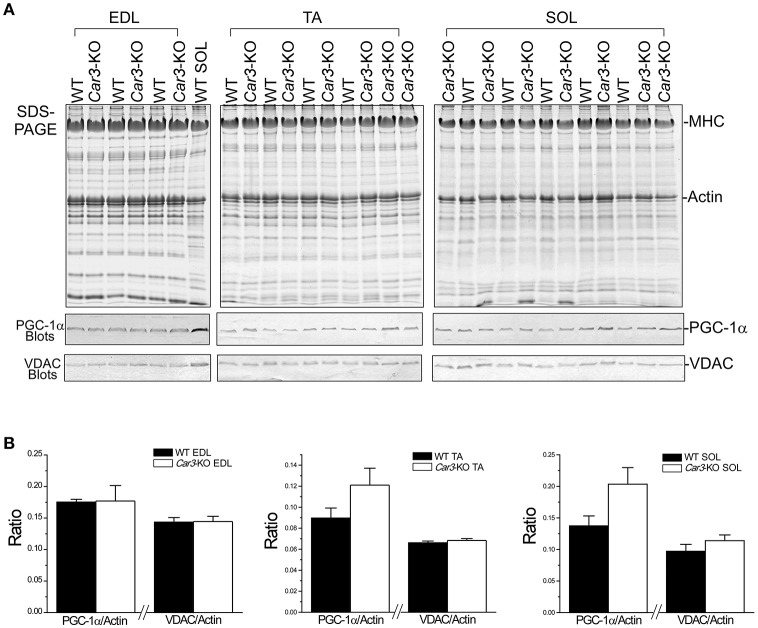
**No changes of mitochondrial markers in ***Car3***-KO skeletal muscles. (A)** SDS-gel and Western blots of WT and *Car3*-KO mouse EDL, TA, and soleus (SOL) muscles using antibodies against PGC-1a and VDAC. **(B)** Quantification of the Western blots showed no significant difference between *Car3*-KO and WT muscles, suggesting no difference in mitochondrial contents and biogenesis. Data are presented as mean ± SE. *N* = 3 in WT and *Car3*-KO EDL groups, *n* = 4 in WT TA and 6 in *Car3*-KO TA groups, *n* = 5 in WT SOL and 7 in *Car3*-KO SOL groups. Statistical analysis was performed using Student's *t*-test.

Western blots for the levels of PGC-1α that participates in the regulation of multiple mitochondrial genes (Hock and Kralli, 2009) and VDAC that represents the total mitochondrial content (Raghavan et al., 2012) showed no significant difference between soleus, EDL and TA muscles of *Car3*-KO and WT mice (Figure 12). The results indicate that the deletion of CAIII did not alter the function of mitochondria in skeletal muscles.

## Discussion

CAIII is present in many skeletal muscles at significant levels but its physiological function remains unclear. Although CAIII is a low activity enzyme among carbonic anhydrases, it is abundant in muscle cells. A previous study has reported unchanged baseline phenotype of *Car3*-KO mice (Kim et al., 2004) which we also used in the present study. The lack of drastic consequences after systemic knockout of the *Car3* gene implicated that CAIII is a nonessential enzyme in the mouse. Since CAIII is a metabolic enzyme that regulates intracellular pH, our study focused on its potential role in muscle fatigue that involves pH changes in the myocytes. Our data present several interesting findings.

### The expression of CAIII in skeletal muscles is not fiber type specific and independent of the expression myofilament protein isoforms

CAIII was thought to have a specific expression in slow fiber rich muscles. However, our data showed significant expression of CAIII in not only slow type but also most of the fast fiber skeletal muscles of adult mouse (Figure 2). Only a few muscles lack CAIII expression, including EDL, masseter and tongue. Therefore, the expression of CAIII in mouse skeletal muscles is not slow fiber specific and independent of the expression myofilament protein isoforms. CAIII has been reported to be under thyroid control (Salvatore et al., 2014). Although thyroid hormone affects muscle fiber type and MHC isoform expression, mice used in our study are of normal thyroid function and the expression of CAIII in mouse muscle is independent of MHC isoforms (Figure 2). Therefore, the possible hormonal influences on the conclusions of our present study merits future investigation.

A hypothesis is that the expression of CAIII in skeletal muscle may correspond to the functional features of the muscle. For example, both TA and EDL are classified as typical fast type muscles but only TA expresses CAIII (Figure 2C). Consistent with a previous report that the expression of *Car3* mRNA starts in all skeletal muscles at early embryonic stage before concentrated in slow fiber muscles (Lyons et al., 1991), our developmental studies showed that CAIII expression was maintained in soleus muscle from neonatal to adult, but ceased 7 days after birth in TA and EDL muscles (Figure 10B). Accordingly, the expression of CAIII in early developmental myoblasts has been suggested as a diagnostic marker for muscle disease (Shima et al., 1983), allowing detection in human fetal plasma samples for diseases such as Duchenne muscular dystrophy (Carter et al., 1982).

An interesting finding is that CAIII is re-expressed in adult TA muscle but not EDL (Figure 10B), suggesting a secondary adaptation that activates *Car3* expression in adult TA muscle in response to a specific functional demand. The expression of CAIII in TA but not EDL muscles was also reported in rat (Shiels et al., 1984). The mechanism of *Car3* gene reactivation requires further investigation. Denervation and innervation affected the level of CAIII in TA and soleus muscles (Milot et al., 1991), supporting a role of the functional feature of muscle in the regulation of *Car3* expression. As a counteractive muscle, TA muscle's activity is related to that of the slow fiber-containing soleus and gastrocnemius muscles. More studies are merited to investigate the role of CAIII in this type of coordinated function of skeletal muscles.

### CAIII increases muscle resistance to fatigue under physiological conditions

Our results showed that CAIII-positive fast muscle TA is more resistant to fatigue than that of CAIII-negative fast muscle EDL in *in situ* contractility studies (Figure 3) and *Car3*-KO TA muscle is less tolerate to fatigue than that of WT TA muscle in *in situ* contractility studies (Figure 6). In the meantime, *Car3*-KO did not change muscle mass and contractile protein isoform contents or baseline twitch and tetanic contractions (Figures 5, 6). Therefore, CAIII does not directly impact on the basic contractility of skeletal muscle but functions in modulating the intracellular environment of muscle cells. The observation that CAIII positive skeletal muscles have higher resistance to fatigue under physiological conditions is a novel finding and indicates a potential mechanism to improve muscle function and durability.

The finding that the loss of slow fiber contents in *Tnnt1*-KO mouse soleus muscle did not cause decrease in CAIII expression (Figure 11) further supports the fiber type independent and functional demand determined expression of CAIII. The maintained high level expression of CAIII may contribute a partial sustention of fatigue resistance in the weight bearing soleus muscle of *Tnnt1*-KO mice. The observation that only a few mouse fast fiber muscles are lack of CAIII, such as EDL, tongue and masseter (Figure 2), suggests that an increase in the level of CAIII may be explored as an anti-fatigue treatment for *TNNT1* myopathies.

Muscle fatigue is known as a decline of muscle performance in repeated and intense muscle contractions. Among multiple processes from excitation-contraction signaling to intracellular ionic equilibrium to changes in metabolites and action of contractile proteins (Allen et al., 2008; Kent-Braun et al., 2012), the fundamental changes during muscle fatigue are the inevitable accumulation of intracellular inorganic phosphate and hydrogen ions which cause impaired functions of Ca^2+^ handling system and contractile proteins. Decreased intracellular pH is one of the factors that produce muscle fatigue during intensive contractions. Intensive contractions also increase metabolic production of CO_2_. By catalyzing the hydration of CO_2_ to H_2_CO_3_, CAIII may play a role in adjusting intracellular pH to counter acidosis in muscle fatigue.

### CAIII increases the sensitivity of muscle to fatigue in acidosis

In contrast to the anti-fatigue function of CAIII *in vivo* under physiological conditions, *Car3*-KO soleus muscle exhibited not decreased but increased resistance to fatigue as compared with that of WT soleus in *ex vivo* fatigue test especially under acidosis condition (Figure 8). Consistent with the anti-fatigue function of CAIII under physiological conditions, *Car3*-KO TA muscle is less tolerating to fatigue than that of WT soleus in the early phase of *in situ* fatigue test (Figure 6C). However, *Car3*-KO TA muscle maintained higher force production in the later phase of *in vivo* fatigue test where local acidosis might have occurred (Figure 6C). This observation suggests that the anti-fatigue function of CAIII is restricted to the physiological range of intracellular pH. A hypothesis is that it functions to decrease the CO_2_ produced in the acute phase of intensive contractions (loss of CAIII causes CO_2_ accumulation and faster dropping of force as shown in Figure 6C). The accumulation of ${\text{HCO}}_{3}^{-}$ produced by CAIII would then decrease intracellular pH and the acidosis would worsen muscle fatigue. Therefore, deletion of CAIII in *Car3*-KO TA muscle actually produces higher remaining force in the later phase of fatigue contractions than that of WT control (Figure 6C). Further experimental studies directly measuring intracellular CO_2_ and pH are required to test this hypothesis.

It is also interesting that *Car3*-KO soleus muscle had a significantly less rise of resting tension in the early phase of fatigue contractions (Figure 9A) corresponding to better sustained force development (Figure 9B). This phenomenon was more predominant at the acidotic condition produced with 30% CO_2_-gassed perfusion buffer. The elevated resting tension may be due to the slower relaxation during muscle fatigue (Westerblad and Lannergren, 1991). Such elevation of resting tension was absent in TA and EDL muscle *in vivo* under physiological conditions with complete relaxation to the baseline during fatigue test (Supplement Figure 2). The mechanism for CAIII to raise resting tension of muscle in fatigue and acidosis remains to be investigated. The rate of sarcoplasmic reticulum Ca^2+^ ATPase (SERCA) pump to uptake Ca^2+^ decreases in muscle fatigue (Allen et al., 1995), which may contribute to elevation of resting tension and lower force development since less Ca^2+^ uptake causes less release during contraction. Low pH inhibits the SERCA pump (Allen et al., 1995; Wolosker et al., 1997). Therefore, our data suggest that the deletion of CAIII in soleus muscle may sustain SERCA activity during soleus muscle fatigue by reducing the productions of ${\text{HCO}}_{3}^{-}$ and H^+^ under preexisting acidosis (Figure 9A). A previous NMR study showed more decreases in phosphocreatine, ATP and pH in *Car3*-KO mouse gastrocnemius muscle following a short period (2 min) of intense contractions than that in WT controls (Liu et al., 2007), further supporting the hypothesis that the functions of CAIII in regulating intracellular pH is dependent on the range of intracellular pH.

### Maintained expression of CAIII in *TNNT1*-KO soleus muscle despite the loss of slow fiber contents

Revising the notion that CAIII expression in skeletal muscle was specific to type I slow-twitch muscle fibers (Shima, 1984; Vaananen et al., 1985; Zheng et al., 1992; Sly and Hu, 1995), we demonstrated that its expression in soleus muscle remains when the slow fiber content was diminished due to the loss of slow TnT in a transgenic mouse model of *TNNT1* nemaline myopathy (Figure 11). Mimicking a recessively inherited lethal disease originally found in the Amish caused by a nonsense mutation at codon Glu^180^ (Johnston et al., 2000; Jin et al., 2003; Wei et al., 2014), the loss of slow TnT in *Tnnt1*-KO mice caused a drastic loss of type I slow fiber contents and a switch to more fast fiber contents in soleus muscle with significantly decreased resistance to fatigue (Wei et al., 2014). The maintained level of CAIII in *Tnnt1*-KO soleus muscle not only demonstrates that the expression of CAIII is not restricted to slow fibers in the soleus muscle but also suggests an adaptation to sustain fatigue tolerance.

The up-regulation of CAIII in fast fibers suggests a novel approach to compensate for the lost function in a sarcomeric myopathy. It is worth noting that the slow isoform of TnI remains detectable in *Tnnt1*-KO soleus muscle (Figure 11A), indicating a maintained slow muscle tissue environment that may play a role in sustaining CAIII expression and a plausible foundation for exploring new treatment of *TNNT1* myopathies.

In summary, our present study demonstrates that CAIII functions in skeletal muscle involving a regulation of CO_2_ metabolism and intracellular pH environment, which contributes to the resistance to fatigue. In the meantime, CAIII expression is not solely dependent on muscle fiber types or developmental stages, but may be determined by functional features of the muscle such as slow muscles and functionally related fast muscles. Representing a novel mechanism to reduce muscle fatigue in physiological conditions and compensate for muscle function in diseases that lose slow fibers, the function and regulation of CAIII in skeletal muscle merits further investigation.

## Author contributions

HF: Performed experiments, designed and modified protocol, drafted the paper, edited text, and figures, approved submission. JJ: Designed research, drafted the paper, edited text, and figures, approved submission.

### Conflict of interest statement

The authors declare that the research was conducted in the absence of any commercial or financial relationships that could be construed as a potential conflict of interest.

## Supplementary material

Supplementary Figure 1 **Young adult ***Car3***-KO mice had normal mass and fiber size in TA and EDL muscles. (A)** 2-month old male *Car3*-KO and WT mice showed similar body weight. **(B)** Normalized to body weight, the weight of *Car3*-KO and WT TA and EDL muscles are nearly identical. **(C)** H&E stained cross sections showed similar fiber cross-sectional areas in WT and *Car3*-KO mouse TA muscles with no signs of degeneration in *Car3*-KO TA muscle. **(D)** Quantification by normalizing to the body weight showed that the cross-sectional areas of *Car3*-KO and WT TA muscles had significant difference. The data are presented as mean ± SE. For **(A,B)**, *n* = 8 mice in WT TA group and *n* = 7 mice in *Car3*-KO TA group; *N* = 5 mice in WT EDL and *n* = 7 mice in *Car3*-KO EDL groups. For **(C,D)**, *n* = 4 mice each in WT and *Car3*-KO groups. Statistical analysis was performed using Student's *t*-test.

Supplementary Figure 2 *****In situ*** fatigability test of mouse TA and EDL muscles showed no increase of resting tension**. The resting tension of WT and *Car3*-KO mouse TA **(A)** and EDL **(B)** muscles during in situ fatigue contractions showed that under *in vivo* physiological systemic pH environment, *Car3*-KO and WT mouse TA and EDL muscles all had no increase in resting tension during fatigue contractions. *N* = 4 in WT TA and WT EDL groups. *N* = 3 in *Car3*-KO TA and 5 in *Car3*-KO EDL groups. Statistical analysis was performed using two-way ANOVA with adjusted mean comparison of Bonferroni test.
