# Supplementary figures and images for: Carbonic Anhydrase III Is Expressed in Mouse Skeletal Muscles Independent of Fiber Type-Specific Myofilament Protein Isoforms and Plays a Role in Fatigue Resistance
Source: Front Physiol. 2016 Dec 15;7:597. doi: 10.3389/fphys.2016.00597 (PMC5156832; doi:10.3389/fphys.2016.00597)

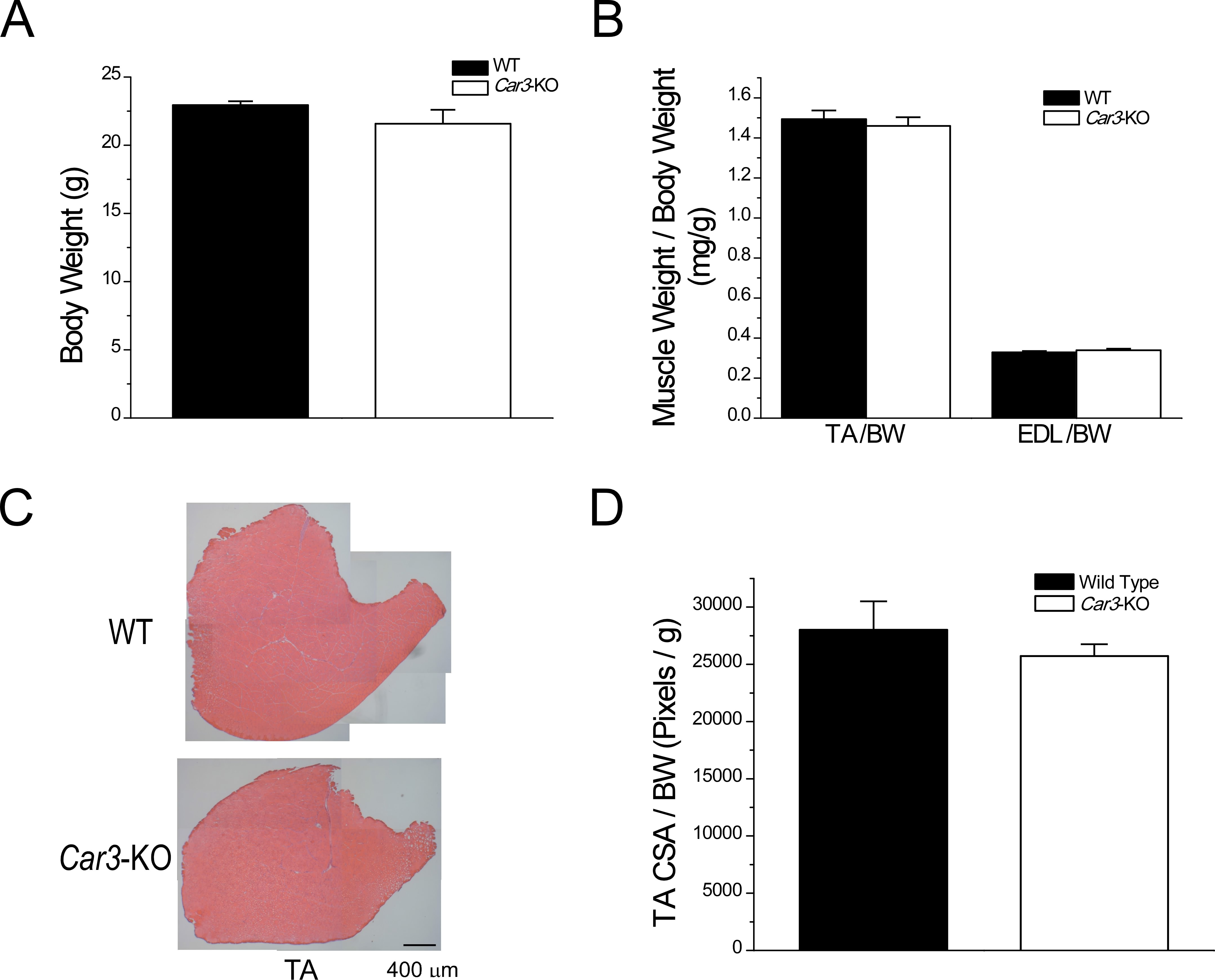

Supplement: Supplementary Figure 1 — Young adult Car3-KO mice had normal mass and fiber size in TA and EDL muscles. (A) 2-month old male Car3-KO and WT mice showed similar body weight. (B) Normalized to body weight, the weight of Car3-KO and WT TA and EDL muscles are nearly identical. (C) H&E stained cross sections showed similar fiber cross-sectional areas in WT and Car3-KO mouse TA muscles with no signs of degeneration in Car3-KO TA muscle. (D) Quantification by normalizing to the body weight showed that the cross-sectional areas of Car3-KO and WT TA muscles had significant difference. The data are presented as mean ± SE. For (A,B), n = 8 mice in WT TA group and n = 7 mice in Car3-KO TA group; N = 5 mice in WT EDL and n = 7 mice in Car3-KO EDL groups. For (C,D), n = 4 mice each in WT and Car3-KO groups. Statistical analysis was performed using Student's t-test. [file Image1.JPEG]

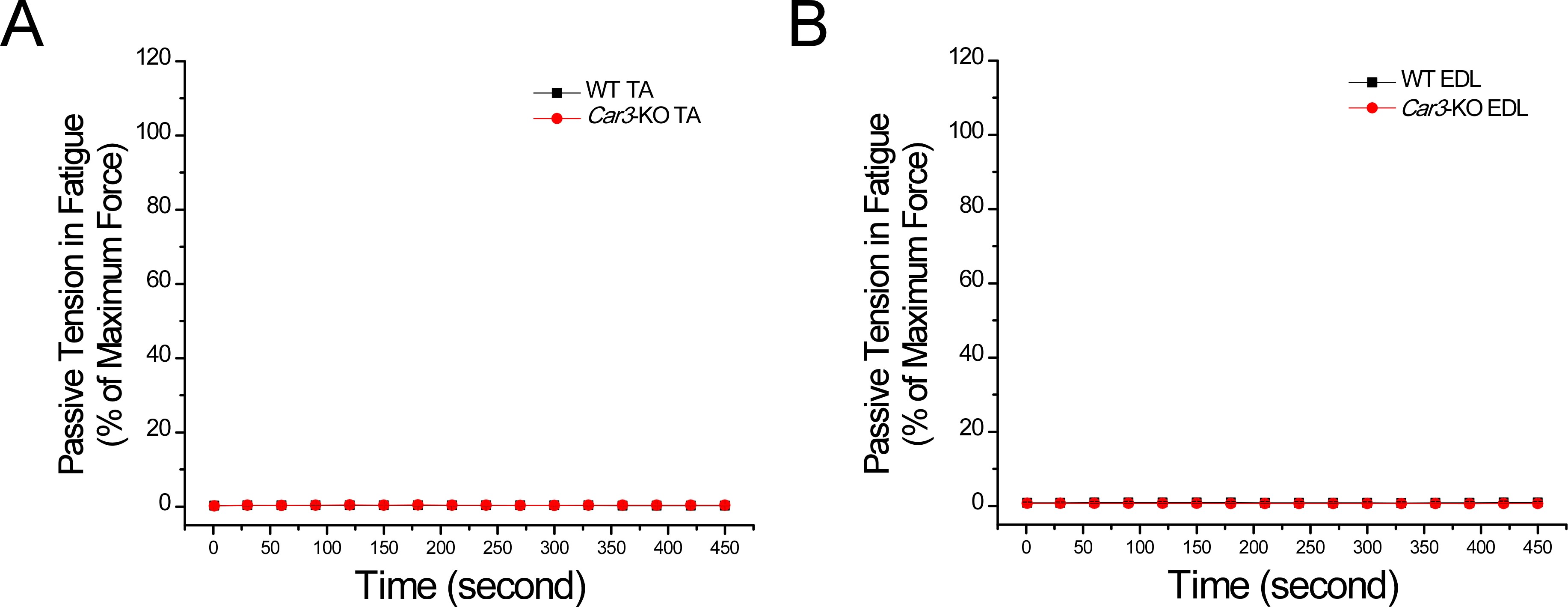

Supplement: Supplementary Figure 2 — In situ fatigability test of mouse TA and EDL muscles showed no increase of resting tension. The resting tension of WT and Car3-KO mouse TA (A) and EDL (B) muscles during in situ fatigue contractions showed that under in vivo physiological systemic pH environment, Car3-KO and WT mouse TA and EDL muscles all had no increase in resting tension during fatigue contractions. N = 4 in WT TA and WT EDL groups. N = 3 in Car3-KO TA and 5 in Car3-KO EDL groups. Statistical analysis was performed using two-way ANOVA with adjusted mean comparison of Bonferroni test. [file Image2.JPEG]
